# Supplementary material for: Are carbon emissions trading and green financial instruments synergistic? -Comprehensive quantitative research based on content analysis
Source: PLoS One. 2024 Mar 7;19(3):e0298601. doi: 10.1371/journal.pone.0298601 (PMC10919601; doi:10.1371/journal.pone.0298601)
Supplement: S1 Data — (ZIP) [file pone.0298601.s001.zip › Data/地级市Z_F工作报告.zip!/║■─╧/║Γ╤⌠╩╨/└┤╫╘╣1⁄2╓┌║┼í╛┬φ┐╦╩2╛▌═°í┐_2014.docx]

各位代表：

现在，我代表市人民政府，向大会作政府工作报告，请予审查，并请各位政协委员和其他列席人员提出意见。

一、2013年工作回顾

过去一年，在市委的坚强领导下，在市人大、市政协的大力支持下，市政府团结带领全市各级各部门，紧紧围绕“提升新标杆、打造升级版”的总要求，加快全面建成小康社会进程，较好地完成了市十四届人大一次会议确定的各项目标任务，实现了本届政府工作的良好开局。

——经济发展稳中有进。切实扭转年初低迷开局，成功战胜历史罕见特大干旱，实现了经济总量、增长速度、发展质量的同步提升。全市地区生产总值突破2000亿元大关，达2171亿元(初步核算，下同)，增长10.5%；财政总收入突破200亿元大关，达211.88亿元，增长13.01%；完成固定资产投资1436亿元，增长35%；实现社会消费品零售总额731.92亿元，增长13.9%。金融机构存、贷款余额分别为2104亿元、915亿元，分别增长17.3%、31.2%，存贷比提升4.6个百分点。

——结构调整步伐坚实。三次产业结构调整为15.5:48.2:36.3，一产占比下降1个百分点，三产占比提高1.3个百分点。规模工业增加值增长11.8%，以电子信息产业为代表的战略性新兴产业产值增长20%；成为全国首批信息消费试点市。粮食生产在大旱之年确保稳产增产，粮食收购量居全省第一，衡南被评为全国粮食生产先进县；油茶产业发展提质提速，我市入选全国油茶产业发展示范市。服务业综合改革深入推进，现代物流、养老服务等5个重点行业服务业标准化试点通过省级立项。我市入列全国二级物流园区布局城市，获批衡阳国家农业科技园区，荣获“全国科技进步先进市”。

——城市建管提速提质。完成中心城区总体规划修改，全面启动各县城总体规划修改工作。衡州大道全线通车，来雁新城、滨江新区等城市地标项目顺利开工。二 环路、船山东路等城区骨干道路建设加快推进，蒸阳北路等6条道路改造完毕，提质改造旧城区15.9平方公里，老城区成熟居民片区改造基本完成。整合提升 “数字衡阳”四大应用平台，扎实推进城市管理“六大战役”、“两大工程”，新增和提质绿化面积51万平方米，基本完成“一江六路九桥”亮化提质任务。大力 开展“规电”、“禁停”等专项行动，城市管理更加规范有序，城市面貌焕发新的容颜。

——社会大局和谐稳定。着力构建信访新秩序，坚持开门接访，健全问题解决和积案化解机制，信访总量、越级非正常上访量、重复非正常上访量明显下降。狠抓 安全生产，煤矿整治整合取得阶段性成效，全年未发生重大及以上安全生产事故，安全指标控制情况为近年来最好的一年，安全生产考核排名全省第一，耒阳、石 鼓、祁东被评为全省安全生产先进县(市)区。大力构建严密的治安防控体系，深入开展严打整治，群众的安全感和满意度明显提升，社会发展大局持续和谐稳定。

一年来，我们坚定不移地致力于打基础、谋长远，求实效、惠民生，在工作重点上做到了“四个突出”。

(一)突出项目建设。创新重点项目板块负责推进机制，全年开工建设重点项目337个，完成投资653.87亿元。交通骨架基本拉开。衡阳南岳机场预计今年上半年试飞通航，湘桂高铁、衡茶吉铁路开通运营，怀邵衡铁路前期工作进展顺利；京港澳高速衡阳段改造、潭衡西匝道交通工程竣工通车，衡云干道建设加速推进，娄衡高速、南岳高速东延线、衡大高速松木互通、武广衡山西站至南岳公路、衡西快速干道开工建设；土谷塘航电枢纽建设扎实推进，松木港(一期)工程投入营运；完 成干线公路前期工作186公里、通畅工程519.6公里，成功创建文明样板路151公里。配套设施明显改善。严格市政工程建设监督管理，开工建设市政工程 项目47个，完成投资66.8亿元。完成污水管网改造90公里，新建和提质改造城镇道路150公里。蒸水南堤风光带(二期)等项目圆满完成，幸福河改造、 江东污水处理厂(一期)等项目先后开工建设，营盘山公园、虎形山公园竣工开园。产业项目支撑有力。中国五矿水口山金铜、华耀城、红星美凯龙等旗舰项目开工 建设，富士康消费电子、金杯电工核电电缆、建滔化工(四期)、中兴网信全球共享服务中心、华菱衡钢热处理生产线等项目竣工投产，特变电工输变电产业园、南 岳生物制药、崇盛国际中心、金甲湖农业科技示范园等项目顺利推进，城区3万亩蔬菜基地基本建成。民生项目扎实有效。2座中型水库、188座小二型水库除险 加固任务全面完成，市体育馆、游泳馆、美术馆、影剧艺术中心、群众艺术中心、青少年活动中心及市中心医院异地新建等项目先后开工建设，南华大学新校区、湖 南高铁职业技术学院新校区(二期)、湖南科技经贸职业学院升本(二期)、湖南师大附中耒阳分校建设顺利推进。

(二)突出改革创新。重点领域改革实现突破。投融资体制改革扎实推进，市本级投融资公司法人治理结构逐步完善，全年到位融资85.6亿元，成功发行16亿元湘江流域重金属污染治理债券。金融改革来势喜人，启动农村金融改革，耒阳、常宁农村商业银行成功挂牌营业；中 油金鸿定向增发募集资金17.9亿元，全市重点上市后备企业达37家。市公交公司股权调整基本完成，城区公交网络持续优化提升。启动45家企业改制，完成 改制28家，安置职工1.37万人。殡葬改革全面启动，市殡仪馆建设基本完成。“两型”社会配套、事业单位分类、水利、文化体制等其他领域改革进展顺利。 机制体制创新步伐加快。进一步理顺市区两级管理职能，切实增强城区自我管理和发展能力。积极推进预算绩效管理工作。试点推行“一流转五服务”为主要内容的 农村综合改革，新发展农民专业合作组织476个，带动农户9.5万户，7个农机合作社荣获全国示范农机合作社。对外开放成效明显。承接产业转移项目503 个，世界500强企业和大型央企均达17家。公路口岸正式运行，衡阳综合保税区高标准通过正式验收并成功运行，开放平台的数量和层次均居全省之首。全年实 际利用外资7.78亿美元，增长28.7%；完成进出口总额18.14亿美元。

(三)突出民生改善。坚持财税收入主要用于民生改善的支出导向，全市民生支出达239.2亿元，占财政总支出的66.5%。城乡居民收入分别达 22450元、11400元，分别增长10.7%、12.7%。守住了环保底线。扎实推进污染减排和污染防治，提前2年完成重金属污染治理两个“50%” 削减目标，全面完成省下达的节能减排任务。农村环境综合整治扎实推进，4个村被评为省级新农村建设示范村。办好了民生实事。20项为民办实事任务圆满或超 额完成。新增城镇就业7.84万人，新增农村劳动力转移就业6.35万人。企业退休人员养老金实现“九连调”，城乡低保人均补差水平分别提高到285元/ 月、123元/月，农村五保分散供养保障标准提高到每人每年2542元，城乡计划生育特别扶助标准提高到每人每年2820元。开工建设保障性住房 18349套，基本建成7921套。解决35.4万农村人口饮水安全问题。抓实了社会事业。完成267所合格学校建设，开工建设417所农村义务教育薄弱 学校。完成“村村通”直播卫星安装15143户，“公共文化服务进社区”被评为国优项目，城市文明创建工作顺利通过省级测评。医药卫生体制改革进一步深 化，新农合参合率达99..7%，基本药物制度实施成果进一步巩固。人口和计划生育工作连续十年保持全省先进。食品安全工作稳步推进。体育事业蓬勃发展， 市体育局荣获全国体育系统先进集体。海关、检验检疫服务外向型经济发展成效突出。工会、共青团、妇联、残联等群团工作取得新的成绩。驻衡解放军、武警部 队、民兵预备役部队作出新的贡献，国防后备力量建设、国防教育和“双拥”工作创新发展。

(四)突出环境优化。全面清理行政审批项目，依法取消、下放、封存行政审批项目超过1/3；大 力推进全程代办制度和网上办理工作，审批提速50%以上。扎实开展“千名干部下企业”活动，帮助企业协调解决主要问题650个。坚持依法行政，自觉接受人 大及其常委会的法律监督和工作监督，自觉接受人民政协和各民主党派、工商联、人民团体、无党派爱国人士的民主监督，一年来办理人大代表议案1件、建议 320件，政协提案491件。坚持用制度管权管事管人，把权力关进制度的笼子里，突出解决“四风”问题，保持查办腐败案件的高压态势，进一步营造宽松优良 的发展环境。

各位代表!过去的一年，我市经济社会发展取得的成绩，是市委正确领导的结果，是市几大家精诚团结的结果，更凝聚了各位人大代表、政协委员及全市人民的心 血和汗水。在此，我谨代表市人民政府，向给予政府工作大力支持的各级人大代表、政协委员和全市人民，向关心支持地方工作的人民解放军驻衡部队、武警部队官 兵和中央、省驻衡单位、高校、科研院所，向为衡阳发展作出贡献的广大投资者和外来务工人员，表示崇高的敬意和衷心的感谢!

各位代表，我们必须密切关注、认真解决经济社会发展中面临的困难和问题。一是发展动力仍然不强。重大项目储备不足，影响衡阳长远发展的战略项目不多；外贸依存度偏低，开放型经济活力有待提升；区域发展不平衡，县域经济缺乏特色支柱产业的支撑，与中心城区之间的经济占比差距越来越大。二是发展方式仍然不优。第一产业占比较高，缺乏主导产业和龙头企业的引领；服务业综合改革试点验收压力较大；企业自主创新成果产业化、资本化水平低；高能耗行业比重较高，资源环境制约日益突出，节能减排和环境治理任务比较艰巨。三是发展效益仍然不佳。财政收入结构不优，税收占财政收入的比重有待提高；城乡居民收入总体水平低、区域差异大。四是行政效能仍然不高。行政审批有待进一步精简提速，“四风”问题未能根除，部门的服务意识和服务态度还有很大的提升空间。对此，我们必须采取有力措施，坚持不懈地加以解决。二、2014年目标任务和主要工作

2014年，是全面贯彻落实党的十八届三中全会精神、全面深化改革的开局之年，也是加快全面建成小康社会和落实“十二五”规划的攻坚之年。当前，国际经济形势更加复杂，面临不少风险和挑战；我国经济处于换档期，下行压力比较大。但全球经济温和复苏，主要经济体保持增长；我国经济发展长期向好的基本面没有变，特别是国家新一轮改革将释放巨大的改革红利和发展活力。我们既要看到有利形势和已有优势，也要警醒发展压力和工作短板，增强忧患意识、机遇意识和责任意识，牢牢把握工作主动权，奋力谱写衡阳改革发展的新篇章。

今年政府工作的总体要求是：全面贯彻落实党的十八大、十八届三中全会和中央、省委、市委经济工作会议精神，围绕打造“工业重镇、文化名城、旅游胜地、宜 居家园”总目标，实施“开放带动、基础先行、兴工强农、创新发展”总战略，坚持稳中求进，推进改革创新，注重统筹发展，着力改善民生，促进经济持续健康发 展、社会和谐稳定，加快全面建成小康社会。

今年全市经济社会发展的主要预期目标是：地区生产总值增长10.5%以上，财政总收入增长14%以上，固定资产投资增长30%，社会消费品零售总额增长 13.5%，进出口总额增长30%，城乡居民收入增长12%，城镇登记失业率控制在4.5%以内，人口自然增长率控制在8.9‰左右，完成省下达的节能减 排目标任务。

实现上述目标，必须坚决贯彻落实以下原则。一要坚持稳中求进。巩固稳中向好的发展态势，在稳的基础上全力求进，统筹稳增长、调结构、促改革，促进经济社 会大局稳定，努力实现经济发展质量和效益得到同步提高的速度。二要推进改革创新。积极推动全面深化改革，坚持问题导向，坚决破除各方面体制机制弊端，发挥 市场在资源配置中的决定性作用，将衡阳打造成为经济运行结构良好的城市。三要注重统筹发展。以全面建成小康社会目标统揽经济社会发展全局，突出分类指导、 协调推进，把工作的重心和重点放在拉长短板上，加快县域经济发展，抓实抓好“三农”工作，更加注重社会建设，更加注重生态环保，统筹城乡区域协调发展。四 要着力改善民生。按照“守住底线、突出重点、完善制度、引导舆论”的思路，坚决守住安全生产、环境保护、社会稳定底线，扎实做好民生工作，确保财税收入主 要用于民生改善，让改革发展成果更多更公平地惠及全市人民。

(一)深化改革开放，增强发展动力活力。以更大的决心和勇气推进改革创新，力争在新一轮对内对外开放中赢得主动。

全面推进重点领域改革。深化财税体制改革。坚持做大、做优、用好财政收入，扩大预算公开范围，逐步建立科学合理的预算绩效管理机制；积极推进“营改增”试点；规范政府性债务举借、使用、偿还行为。深化金融体制改革。规范和加强政府投融资公司建设，力争年内到位融资100亿元；构建企业直接融资体系，积极探索新型融资模式，力争年内实现社会融资300亿元；加快发展地方性金融组织，深化农信社改革发展，加快推进城区信用联社组建市农商行步伐，加大村镇银行组建工作力度，规范发展融资性担保公司和小额贷款公司；加 强金融监管，推动金融诚信建设，打造金融安全区，积极争创全省金融生态良好城市。扎实推进国企改革。坚持“先安置、后处置”，引进战略投资者、鼓励非公有 制企业参与国企改革，支持企业做大做强。深化市公交公司改革。优化和拓展公交线路，提升公交服务质量，打造让市民基本满意的公交系统。加紧推进殡葬改革。 完善基本殡葬公共服务设施及网络，落实惠民殡葬政策，积极倡导殡葬新风。

大力推进体制机制创新。进一步理顺市区两级管理职能，推进环卫、绿化管养、风光带管护市场化改革，理顺区园关系，稳步下放教育、卫生等社会职能，赋予城 区更多的自主权。实施科技创新驱动战略，健全科技创新投入机制，发挥市场对创新要素配置的导向作用和企业在技术创新中的主体作用；扩大在衡高校教育资源优势，培养、引进高层次、高技能和实用人才。推进应用型技术研发机构市场化、企业化改革，发展技术产业交易市场，力争全年实现高新技术产业产值1300亿元。

着力扩大对内对外开放。抓紧完善衡阳综合保税区各个专项规划和配套设施，支持“无水港”、公路口岸、铁路口岸、航空口岸等开放平台的运营和成长，促进口 岸经济跨越发展。大力扶持重点出口企业发展壮大，鼓励传统优势产品和具有自主知识产权的高科技产品扩大出口，加大高新技术、成套设备进口，努力增加开放型 经济对经济增长的贡献份额。加强重大项目深度开发及策划包装，加快承接产业转移，加大力度引导衡商回衡发展，深化与央企的对接合作，吸引更多企业来衡建立 集团总部、区域总部和研发、采购中心，提高开放型经济发展水平。

(二)加快结构调整，促进产业转型升级。深入开展“项目建设年”活动，大力实施400余个、年度投资800亿元以上的重点项目建设计划，以项目拉动投资，助推产业转型升级。

着力推进新型工业。改造提升传统优势产业。围绕打通完善工业产业链，深度开发“盐卤、有色、瓷泥、煤炭、铁矿”等资源加工产业，推进汽车零部件、盐卤化 工、有色金属、输变电、钢铁冶炼等传统主导产业转型升级，加快中国五矿水口山金铜、安徽华菱天然气重卡、深圳中航标准件、南岳电控等项目建设，实现规模效 益倍增。着力发展战略性新兴产业。推进电子信息、生物医药、新材料、新能源等战略性新兴产业的技术研发，加速科技成果产业化、资本化，重点推进鸿富锦精密 模具、共创光伏(二期)、南岳生物制药、恒生制药、湖南金叶烟草薄片等项目建设，支持富士康、中兴网信、欧姆龙、中科光电做大做强，力争信息产业产值增长 20%。大力实施品牌战略。实施工业龙头企业培育工程和中小企业“百千万”成长工程，引导重点企业兼并重组，加大技术改造力度，建立技术(工程)中心、科 技创新(成果转化)产业基地，参与国家标准制定，争创中国驰名商标。加快工业园区建设发展。实施省级工业园区建设提质及产业倍增计划，推进省级及以上产业 园区调规扩区工作，加快特变电工产业园、衡钢加工园(二期)建设，依靠龙头企业分类带动产业向市直两区一园集聚，力争园区规模工业增加值占全市规模工业增 加值的比重达37%以上。

大力发展现代农业。加快实施农业综合开发及国土整理项目，大力推进高标准农田建设，稳定粮食耕种面积，扩增地方粮食收储规模，确保粮食总产稳定在330 万吨以上。整合各项涉农资金，加强农田水利建设，加快病险水库除险加固(二期)、骨干山塘清淤扩容和衡邵干旱走廊衡阳区域综合治理、中小河流治理，建设以 “五小水利”、灌区续建配套为重点的农业灌溉体系。完善农业产业体系，抓好畜禽、油茶、林木、果蔬、饲料等特色产业，加快百里油茶产业示范带和各县 (市)8大特色蔬菜基地建设。支持创建农业科技创业园区和县城所在地设立农产品加工园区，积极推进衡阳国家农业科技园区建设。推进农业科技创新，加强农业 品牌建设，大力实施农业标准化，不断提高我市农产品品牌总体实力。注重农产品生产源头治理和产销全程监管，确保广大人民群众“舌尖上的安全”。

加快提升现代服务业。对照“国家服务业综合改革试点城市”2015年验收的时间表和路线图，加快改革试点步伐，促进服务业转型升级。引进和新设各类金融 机构5家以上，着力打造区域性金融服务中心。建好华耀城、商业步行街、崇盛国际中心、红星美凯龙等服务业标杆项目。完善现代物流基础设施，大力发展第三方 物流，加强物流专业化、标准化、信息化建设，有序推进白沙物流园、衡缘物流中心建设，提升现代商贸物流功能。加快“万村千乡市场工程”、“百城千镇县乡流 通再造工程”、农产品直销店建设，积极推进“农超对接”、“农商对接”等活动，支持农贸市场标准化提质改造。对接大湘南旅游规划，围绕打造“大南岳旅游经 济圈”，把衡阳建成具有国际影响力的旅游目的地城市，加快南岳天子山火文化公园、南岳旅游休闲观光园、丽波国际酒店等项目建设，力争全年接待国内外游客、 旅游收入分别增长13%和18%。

(三)对标一流城市，加强城市建设管理。对标全国一流水平，扎实推进城市建设管理，积极创建全国文明城市。

完善城市交通枢纽功能。全方位拓展衡阳的外部交通，确保衡阳南岳机场上半年试飞通航，重点抓好怀邵衡铁路、娄衡高速公路、南岳高速东延线、湘江土谷塘航 电枢纽工程等项目建设。力争年内开工建设湘江株洲至衡阳两千吨级航道、祁东归阳千吨级码头。加快衡云快速干道、衡西快速干道、322国道城区段和武广衡山 西站至南岳公路建设步伐，进一步完善“一小时经济圈”。围绕发挥路网整体效益，加强县(市)区之间的交通衔接，抓好国省干线公路和农村公路建设，进一步巩 固区域性交通枢纽城市地位。

夯实城市基础设施。围绕“旧城提质、新区开发”的建设思路，完善城镇道路、公共交通、供水、供气、污水和垃圾处理、配电网、地下管网等市政设施，重点推 进来雁新城、滨江新区、东洲岛片区、酃湖片区建设，完善白沙洲工业园、松木经开区、华耀城片区路网，加快建设二环路、船山东路、长丰大道北延、蒸湘北路延 伸等7条城市主干道，提质改造东风南路、107国道城区段等重要干道，开工建设耒水西岸、湘江西岸风光带(二期)，加快虎形山公园(二期)、南湖公园、植 物园、酃湖公园建设，全力打造雁城最美名片。抓好幸福河改造，加快珠晖水厂、江东污水处理厂、城市生活垃圾焚烧发电厂及中心城区截污截流管网工程建设。

提升城市管理水平。探索制定城市管理分项标准，加强市政公共设施统筹管理，进一步健全和完善城市管理机制。深入开展“城市管理年”活动，继续推进城市管 理“六大战役”、“两大工程”，推进城市管理精细化、长效化。严格规范村民建房行为，坚决控制城市规划区内的非法用地、违法违规建设。加强对安置房的建设 管理。完善“数字衡阳”配套建设，拓展让市民参与城市管理的渠道。

加强城市资本经营。积极探索市场化建设管理城市的新机制，抓好城市公共设施和无形资产经营管理，实现城市资产增值，合理解决“钱从哪里来”和“钱要怎么 还”两大问题，保持城市建设管理良性化负债。加大土地报批、收储、出让力度，实行园区工业项目用地储备制度。严格规划控制土地市场，盘活存量土地，加强土 地项目包装与资源整合，最大限度提高土地的整体效益。引导房地产市场及相关产业健康发展，加大普通商品房供给。

(四)做强县域经济，统筹城乡和区域协调发展。支持和引导各县(市)发展特色经济，走新型城镇化道路，提升县域经济整体水平。

大力发展县域经济。各县(市)要按照“因地制宜、优势优先”的要求，整合优势资源，引进战略投资者打造1-2个主导产业，构建主业突出、特色鲜明、优势 互补的产业布局。做活、做通、做透产业链条，加快培育龙头企业、专业市场和中介组织，实现单个项目向集群化、链式化发展，打造产业集聚区。强化对各县 (市)工业园区建设发展的量化考核，加快基础设施和配套设施建设，狠抓招商引资和项目落地，不断增强县域经济发展后劲。

积极推进新型城镇化。强化县乡规划，加快县城和小城镇建设，推动中心城区与周边城镇协调、融合发展，打造西渡、南岳、云集、大浦等一批特色卫星城。推进 产业和城镇融合发展，培育建设一批产业大镇、经济强镇、区域重镇和物流商贸、文化旅游特色名镇。全力做好第二批全国重点镇申报工作，每个县(市)至少打造 1个特色城(集)镇。加强传统古镇、村落保护开发。坚持不懈推进新农村建设，不断完善示范片、示范村的基础设施。推进城镇基本公共服务常住人口全覆盖，把 进城落户农民完全纳入城镇住房和社会保障体系，加大农村富余劳动力转移力度，有序推进农业人口向城镇转移。

加快统筹城乡一体化。推进城乡要素平等交换和公共资源均衡配置。建立城乡统一的建设用地市场，推进农村集体经营性建设用地与国有土地同等入市、同权同价；保障农民工同工同酬；完善农业保险制度，推行政府担保和农户联保，鼓励社会资本投向农村建设；推动教育、医疗、社保、文化等公共服务资源向农村地区倾斜。扎实开展“一流转五服务”工作，依法维护农民土地承包经营权，加快推进农村土地承包经营权确权登记颁证工作；鼓励农民采取转包、租赁、互换、转让、入股等多种形式流转土地承包经营权，探索建立农民以承包经营权入股发展农业产业化经营，加快构建以家庭经营为基础，集体、合作、企业经营竞相发展的新型农业经营体系。

(五)建设生态文明，加快构建两型社会。紧紧围绕建设宜居衡阳的目标，建设资源节约型、环境友好型社会。

扎实推进节能减排。强化综合性调控，落实差别电价和惩罚性电价政策，完成淘汰落后产能任务，逐步化解产能过剩。狠抓煤矿、冶炼、化工、电力、水泥、建筑 和交通等重点行业和企业的节能减排，鼓励和支持风能、生物质能、太阳能等可再生能源发展，加大清洁低碳能源产品的推广力度。实行最严格的环境准入制度，落 实污染物总量前置审批制度，没有总量来源的项目一律不予审批，不符合产业政策和环保准入的项目一律不予立项。积极发展循环经济，全面推进松木经开区国家级 循环化改造和湖南机油泵国家级再制造试点，加快松柏、松江、大市、大浦等循环经济园区建设。

铁腕整治环境污染。扎实推进湘江流域保护和治理第一个“三年行动计划”，抓好湘江流域重金属污染治理，关停治理超标排污的工业企业、矿山企业、规模化畜 禽养殖场。加快城镇垃圾、污水处理设施建设。强力推进山体修复和“矿山复绿”行动，加快耒阳市煤矿、常宁市水口山铅锌矿、衡阳县界牌瓷泥矿、衡山县白果石 膏矿等重点老矿区地质环境恢复治理工程建设，加强无主尾矿库隐患综合治理。加强河道管理，坚决杜绝中心城区挖山填水行为。制订大气污染防治计划和应急预 案，大力推广清洁能源，全面淘汰燃煤小锅炉，加强机动车污染防治，扎实开展渣土管理和垃圾、秸秆焚烧专项整治行动。坚持以集镇为中心，全面推进农村环境综 合整治，抓实暴露垃圾清理、水体污染治理、马路市场取缔与搬迁，彻底解决集镇“环境脏、设施差、秩序乱”现象。

加强生态建设保护。加强森林资源保护管护，继续实施人工造林增绿工程，全面推进高速公路、高速铁路、城市外环线、主要国省道、湘江及其一级支流沿线的造 林绿化工作，力争年内城市建成区绿地率、绿化覆盖率分别达33.9%、37.2%，全市森林覆盖率达42%。坚守耕地红线，落实最严格的耕地保护制度和节 约用地制度，严格用途管制，强化节地考核。实行最严格的水资源管理制度，建立和完善用水总量控制、用水效率控制、水功能区限制纳污“三条红线”指标体系和 考评机制，加强集中式饮用水源地保护，确保群众饮水安全。

(六)突出公平公正，让群众共享改革发展成果。着力为民办实事、解难题，推动改善民生制度化、长效化、常态化。

抓好就业创业工作。支持高就业企业和产业发展，帮助企业解决用工结构性短缺问题。实行更加积极、灵活的就业政策，抓好高校毕业生、农村转移劳动力、城镇 困难人员和退役军人就业，实施离校未就业高校毕业生就业促进计划，做好企业下岗工人再就业工作，力争新增城镇就业7万人，新增农村劳动力转移就业6万人， 城镇登记失业率控制在4.5%以内，“零就业”家庭动态就业援助100%，失业人员再就业4万人。落实高校毕业生、科技人员、返乡农民工创业政策，扎实推 进全民创业行动计划，争创省级创业型城市。

完善社会保障体系。加快社会保险体系建设，继续做好城乡居民养老保险征缴扩面工作，全面落实企业退休人员养老金“十连调”。大力推进医保付费方式改革， 稳步推进医疗保险城乡统筹、市级统筹和居民医疗门诊统筹。探索将被征地农民纳入城镇养老保险体系，力争年度征地项目涉及的被征地农民参保率达到80%，已 参保享受待遇支付率达到100%。科学调配住房公积金增值收益和国土出让收益用于保障性住房建设，扎实做好棚户区改造工作，开工建设保障性住房26272 套，确保中低收入家庭、新就业和进城务工人员公平享受住房保障。健全社会救助体系，完善特困群众生活救助物价上涨动态补贴机制，提高基本养老服务补贴覆盖 率。

努力增加居民收入。全面落实强农惠农政策，支持发展村级集体经济，多渠道增加居民财产性收入。健全工资决定和正常增长机制，改革机关事业单位工资和津贴 补贴制度，完成事业单位实施绩效工资扫尾工作。规范收入分配秩序，努力缩小城乡、区域、行业分配差距。推进精准扶贫和产业扶贫，重点抓好产业发展、健全公 共服务和教育三件大事，推动水、电、路、气、房、环境整治“六到户”。切实抓好移民后扶工作，推进移民安置地和谐发展。

(七)夯实社会事业，提升基本公共服务水平

不断深化精神文明和文化建设。深入开展中国特色社会主义和“中国梦”宣传教育，培育和践行社会主义核心价值观。深化群众性精神文明创建活动，推进社会诚 信体系建设。完善文化管理体制，支持转企文化单位发展壮大，探索建立文化馆、图书馆、博物馆法人治理结构。加快推进重点文化惠民工程建设，鼓励新建文化社 团组织，完善公共文化阵地服务体系，大力推进“三馆两中心”建设，积极发展新闻出版、广播影视、文学艺术和档案事业。加强文化遗产保护、利用和传承。加快 文化产业发展，培育新型文化业态，建设文化产业基地。

着力发展社会事业。坚持优先发展教育事业，完善城区中小学及农村义务教育阶段学校布局规划调整工作，努力提升教育教学质量；巩固和发展校舍安全工程建设成果，加大农村薄弱学校改造力度，确保校园安全和校车安全；抓 好南华大学新校区、湖南高铁职业技术学院、湖南师大附中耒阳分校建设和铁一中、市九中搬迁工作。扎实推进医药卫生体制改革，完善新农合制度，巩固基本药物 制度实施成果，积极探索县级公立医院改革试点，加快市中心医院异地新建、南华大学附属南华医院门急诊医技住院综合大楼、全市医疗卫生服务体系建设。坚持计 划生育基本国策不动摇，促进人口长期均衡增长。深入开展全民健身运动，积极备战省第十二次运动会，促进群众体育和竞技体育全面协调发展。健全农村留守儿 童、妇女、老年人关爱服务体系，完善残疾人权益保障、困境儿童分类保障制度。发展公益慈善事业。

切实创新社会治理。加快城乡社区网格化管理进程，做好社区矫正工作，增强城乡社区服务功能和自治能力。坚持以群众工作统揽信访工作，推行网上信访，健全 及时就地解决群众合理诉求机制，强化法律在化解矛盾中的权威地位，建立涉法涉诉信访依法终结制度，进一步完善大信访格局。健全重大决策社会稳定风险评估机 制，依法加强虚拟社会动态管理和网络舆情引导，防范土地动拆迁等可能引发的群体性事件风险，完善应急管理机制体制，提升突发事件应对和处置工作水平。持之 以恒抓好安全生产，强力开展食品药品安全整治，巩固煤矿整治整合成果，继续保持“打非治违”的高压态势，进一步提高企业本质安全水平，坚决杜绝发生重大安 全事故。深入推进社会治安综合治理，推动警力下沉，加快打防管控一体化社会治安防控体系建设，加大毒品问题、交通违法行为专项整治力度，保障人民生命财产 安全。创新军民融合发展方式，加强国防动员、民兵预备役、人防工作，重视军事设施保护，加大全民国防教育力度，积极开展“双拥”共建活动，深入推进国防和 后备力量建设。

(八)加快职能转变，不断增强政府公信力和执行力

推进简政放权。深化行政审批制度改革，接好放好中央、省里下放的审批事项，确保不截留、不梗阻；尽 快列出市一级的权力清单，迅速“三堂会审”，只要有利于管理、有利于提高办事效率、有利于加快发展，就要大胆改、彻底放，最大限度减少政府对微观事务的管 理。正确履行政府职能，切实把政府职能的重点转移到公共服务、市场监管、社会管理、环境保护上来，坚决把政府该做的、要管的事情抓好、抓到位，通过统一规 范的制度设计，为各类市场主体创造开放透明、公平竞争的发展环境。加强政府管理创新，改进和完善政府监管督查和绩效评估，进一步增强政府工作的执行力。

坚持依法行政。自觉维护法律权威，规范行政决策程序，严格按照法定权限和程序履职用权；建立权责统一、权威高效的行政执法体制，不断加大重点领域执法力度，推进行政执法规范化；主动接受市人大的法律监督、工作监督，市政协和各民主党派的民主监督，自觉接受群众监督和舆论监督，加强审计监督；畅通民主渠道，健全基层选举、议事、公开、述职、问责等机制，做好第九次村委会换届选举工作。

切实转变作风。扎实开展党的群众路线教育实践活动，健全领导干部带头改进作风、深入基层调查研究机制，完善直接联系和服务群众制度。推进政务公开，认真 执行《党政机关厉行节约反对浪费条例》，严格执行党风廉政建设责任制和廉洁从政有关规定，着力解决“四风”问题。不断优化政府机构设置和职能配置，按照本 届政府任期内财政供养人员只减不增的原则，严格控制机构编制总量；健全严格的财政预决算和审计制度，坚持勤俭办一切事情，从今年起，全市行政经费“零增长”，“三公经费”只减不增，努力建设廉洁高效政府。

各位代表，党的十八届三中全会吹响了全面深化改革的号角，开启了中华民族伟大复兴的圆梦征程。让我们在市委的坚强领导下，团结带领全市人民，解放思想，开拓进取，真抓实干，为加快全面建成小康衡阳而努力奋斗!
